# Synergistic effects of nifedipine and indomethacin in tocolysis: A translational study

**DOI:** 10.1002/ijgo.70335

**Published:** 2025-06-21

**Authors:** Lucile Yart, Marie Cohen, Begoña Martinez de Tejada

**Affiliations:** ^1^ Department of Woman, Child and Adolescent University Hospitals of Geneva Geneva Switzerland; ^2^ Department of Pediatrics, Gynecology and Obstetrics Faculty of Medicine, University of Geneva Geneva Switzerland

**Keywords:** indomethacin, nifedipine, preterm birth, synergy

Preterm birth (PTB), defined as delivery between 20 and 37 weeks of gestation, is one of the leading causes of neonatal morbidity and mortality worldwide. Current treatment of preterm labor (PTL) involves the use of tocolytic agents, aiming to inhibit uterine contractions by either targeting pathways involved in smooth muscle contraction or promoting smooth muscle relaxation. However, currently available tocolytic agents have short‐term efficacy and do not prevent delivery for more than 7 days.^1^ Furthermore, maintaining or repeating tocolysis to further delay delivery does not seem to be beneficial for the mother or the fetus,^2^ and hospitalization is often required because tocolytic treatments are mainly administered intravenously and it to allows monitoring of potential adverse effects.

The short‐term efficacy of current tocolytic therapies could be explained by their activity being limited to symptomatic treatment of PTL, by inhibiting uterine contraction without treating the cause. Many factors are known to trigger PTL (stress, smoking, hypertension, obesity, ethnicity). However, inflammation seems to be a central effector of labor induction as 40% and 70% of late (from 34 weeks) and early (before 34 weeks) PTB, respectively, are associated with uterine inflammation.^3^

Combination therapies that simultaneously target both the cause of myometrial contractions and cell contraction itself have emerged as a promising approach to enhance tocolytic effectiveness. Previous studies raised skepticism regarding the superiority of combination tocolytics,^4^, ^5^ but clinical trials have demonstrated that combining nifedipine, a calcium‐channel blocker, with indomethacin, a prostaglandin inhibitor, yields superior tocolytic efficacy compared with monotherapy with either agent alone.^6^, ^7^

In this translational study, we evaluated the combined effects of nifedipine and indomethacin on oxytocin‐induced human myometrial contractions using a previously described non‐inflammatory ex vivo model.^8^ Myometrial samples were obtained from 17 term, non‐laboring women undergoing elective cesarean delivery after obtaining informed consent and ethical approval from May to August 2020. Participants delivered healthy singleton infants between 37 and 40 (average 38.5 ± 0.2) completed weeks of gestation. Women with onset of labor, multiple pregnancies, medication for PTL earlier during pregnancy, infectious diseases, or HIV, hepatitis B virus, or hepatitis C virus infection were excluded. We used uterine biopsies from term pregnancies without medical complications for several reasons. First, preterm cesarean deliveries are rarely elective and, when performed, are typically indicated for underlying complications such as pre‐eclampsia or intrauterine growth restriction—both of which may affect the decidua and confound the interpretation of myometrial contractility. Moreover, we deliberately chose not to approach these patients for participation, because they were already managing medical complications and significant emotional stress. Second, for safety and clinical relevance, we excluded patients with clinical infections, as clinical chorioamnionitis is a contraindication for tocolysis. Third, sample processing had to be conducted during regular working hours, which is rarely compatible with urgent or emergency preterm deliveries.

Oxytocin (10 nM) was used to induce ex vivo myometrial contractions, followed by treatment with incremental doses of nifedipine or indomethacin alone or in combination.

Nifedipine significantly reduced contraction force, amplitude, and frequency at concentrations from 0.1 μM (Figure 1a). As expected in a non‐inflammatory model, indomethacin failed to alter contractility at any tested concentration (Figure 1a). However, combination of a suboptimal concentration of nifedipine (0.01 μM) with increasing doses of indomethacin (from 0.01 to 1 μM) significantly reduced contraction amplitude compared with nifedipine alone (Figure 1b).

**FIGURE 1:**
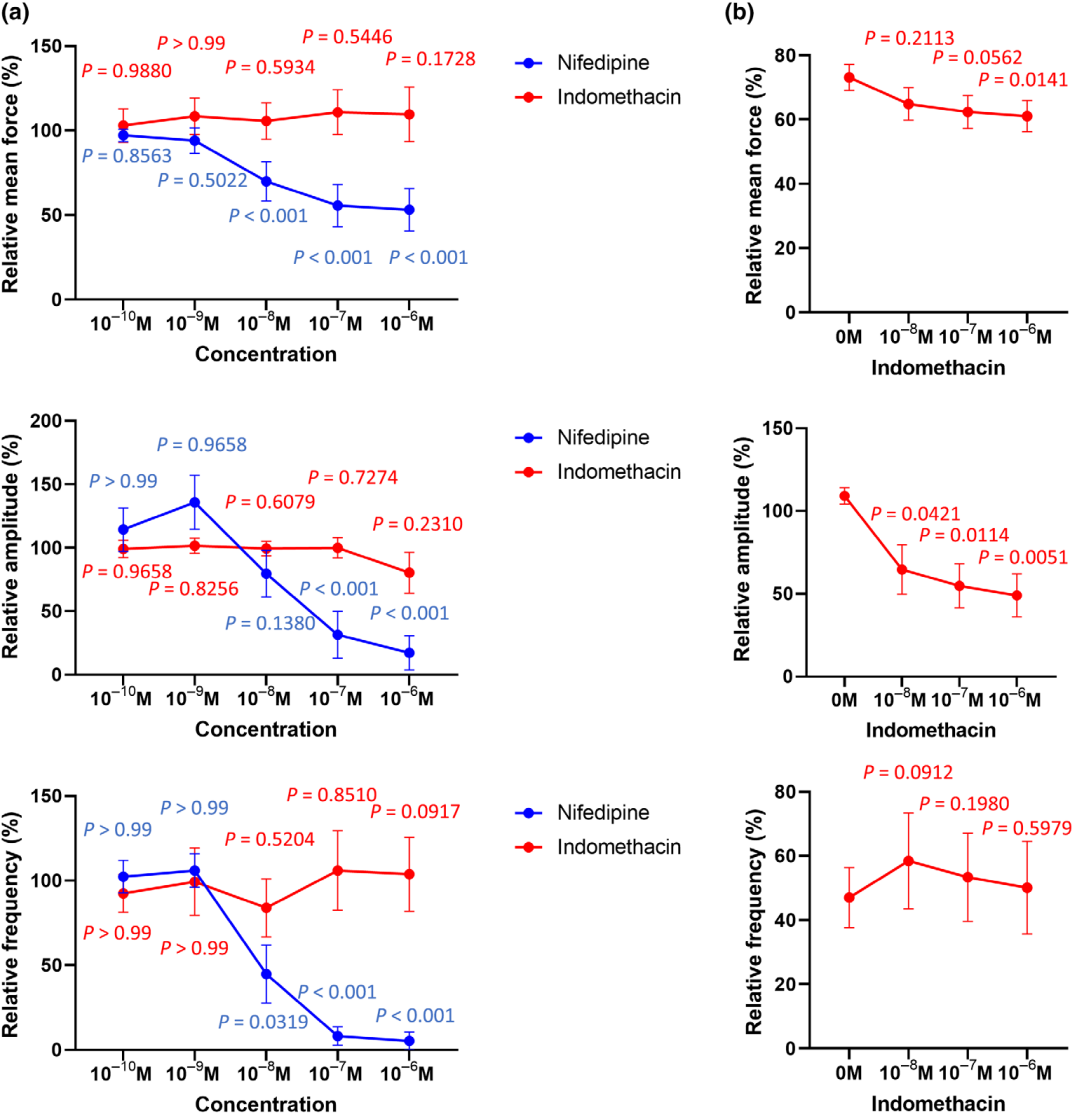
Effect of nifedipine and indomethacin alone (a) or in combination (b) on human myometrial contractions. (a) Effect of nifedipine and indomethacin alone on oxytocin (OT)‐induced human myometrial tissue contraction mean force (area under the curve), amplitude, and frequency. Myometrial strips were treated with increasing doses of each drug from 10^−10^ to 10^−6^ M. (b) Effect of dual combination of nifedipine 10^−8^ M with indomethacin from 10^−8^ to 10^−6^ M on OT‐induced human myometrial tissue contraction mean force, amplitude, and frequency. Data are expressed as percentage calculated from OT‐treated strips. Statistical differences were assessed by analysis of variance.

These findings suggest that indomethacin potentiates the tocolytic effects of nifedipine, even in the absence of an inflammatory context. By enabling the use of lower nifedipine doses, this combination could minimize the adverse effects associated with higher doses of calcium‐channel blockers.

The use of indomethacin as a tocolytic is generally not recommended beyond 32 weeks of gestation because of the potential fetal side effects. However, our findings provide a mechanistic basis for exploring novel combinations that may enhance efficacy, reduce dosage, and improve safety—especially if used earlier in gestation or in a targeted, short‐term manner. Identifying new strategies to improve current tocolytic approaches remains a critical priority.

Our model lacks inflammatory mediators, but our results provide a mechanistic rationale for further clinical investigation of the indomethacin and nifedipine synergy on myometrial contractions. The combination of nifedipine and indomethacin may represent a promising therapeutic strategy to improve current tocolytic approaches and optimize outcomes for patients at risk of PTB.

## AUTHOR CONTRIBUTIONS

LY, MC, and BMT conceived the study; LY performed the experiments; LY and MC wrote the brief communication; and BMT revised and gave the final approval of the manuscript.

## CONFLICT OF INTEREST STATEMENT

The authors have no conflicts of interests.

## Data Availability

The data that support the findings of this study are available from the corresponding author upon reasonable request.
